# Supplementary material for: Perception and practices during the COVID-19 pandemic in an urban community in Nigeria: a cross-sectional study
Source: PeerJ. 2020 Sep 23;8:e10038. doi: 10.7717/peerj.10038 (PMC7519720; doi:10.7717/peerj.10038)
Supplement: Supplemental Information 2 [file peerj-08-10038-s002.pdf]

Inform Consent form

Knowledge, attitude, and practices of community members regarding COVID-19 in Oyo State

Dear respondent,

My name is ____. I am part of a group of researchers from the University of Ibadan. In response to the outbreak of COVID-19 in Nigeria and as part of the government effort to curtail the spread of the disease, we are conducting this study to assess the knowledge, attitude, and practices of community members regarding COVID-19 in Nigeria. The result of the study will be used to guide the development and dissemination of appropriate public health messages that will address knowledge gaps, correct misconception, and improve practices that will halt further spread of COVID-19.

We kindly request you to respond to the following questions to the best of your knowledge. Be assured that all your responses will be treated with utmost confidentiality. Your participation in the study will be highly appreciated.

You are free to refuse to take part in the study. You have a right to withdraw at any giving time if you choose to.

Now that the study has been well explained to you and you fully understand the content of the process, are you willing to take part in the study?

1. Yes 2. No

If no give reasons ____

Signature/ thumbprint of participant………………….. Interview date ……………………

Questionnaire

Knowledge, attitude, and practices of community members regarding COVID-19 in Oyo State

Name of Interviewer<br>LGA<br>Name of street		Date of interview *(dd/mm/yyyy)*		
**Sociodemographic characteristics**				
**1.Age** *(last birthday):*		**2. Sex** 1 M 2 F		
**3. Occupation:**				
**4. Religion:**	1 Christian	2 Islam	3 Traditional	4 Others
**5. Highest level of education:**	1 None	2 Primary	3 Secondary	4 Tertiary
**6. Ethnic group:**				
**Knowledge of COVID-19**				
**7. Have you ever heard about COVID-19?**			1 Yes	2 No

8. What causes COVID-19? *(Do not read options, tick what is mentioned. Multiple responses allowed)*

1. From infected animals to man
2. Touching blood, urine, stool, or saliva from a person who is sick with COVID-19
3. Through insect bites
4. Through spiritual attack
5. Sharing sharp objects such as razors, needles etc with a person who has COVID-19
6. Contact with a person who is sick of COVID-19
7. Contact with beddings, clothing and other personal utensils (plates, cups) of a person who is sick of COVID-19
8. Through the air
9. Participating in burial rites of a person who has died from COVID-19
10. I don't know
11. Other ways *(please specify)*

9. How can COVID-19 be spread? *(Do not read options, tick what is mentioned. Multiple responses allowed)*

1. From infected animals to man
2. Contact with a person who is sick of COVID-19
3. Touching blood, urine, stool, or saliva from a person who is sick with COVID-19
4. Contact with beddings, clothing and other personal utensils (plates, cups) of a person who is sick of COVID-19
5. Through insect bites
6. Through the air
7. Through spiritual attack
8. Participating in burial rites of a person who has died from COVID-19
9. Sharing sharp objects such as razors, needles etc with a person who has COVID-19
10. I don't know
11. Other ways *(please specify)*

10. What are the signs of COVID-19? *(Do not read options, tick what is mentioned. Multiple responses allowed)*

1. Fever
2. General feeling of unwell
3. Weakness
4. Headache
5. Sore throat
6. Abnormal bleeding from any part of the body
7. Rash on the body
8. Body pains
9. Vomiting *(with or without blood)*
10. Diarrhoea *(with or without blood)*
11. Others *(please state)*
12.I don't know

11. When do signs of illness begin after the COVID-19 enters the body?

1. Less than 1 day
2. Between 1 and 14 days
3.More than 14 days
3. I don't know

12. Is there a specific drug/remedy to treat COVID-19?

1. Yes
2. No
1. I don't know

(If yes) What is the drug?

13. Is there a specific vaccine to treat COVID-19?

1.Yes
2. No
3. I don't know

(If yes) What is the vaccine?

14. How can you prevent yourself from contracting COVID-19? *(Do not read options, tick what is mentioned. Multiple responses allowed)*

1. By not touching a person with suspected COVID-19
2. By staying at home
3. Regular handwashing with soap and water
4. Regular handwashing with ash
5. Regular handwashing with water only
6. Regular use of hand sanitizer
7. Drinking salt water
8. Eating bitter kola
9. Avoid eating bushmeat
10. Going for special prayers
11. Bathing with salt water
12. By not touching a person with suspected COVID-19
13. By not participating in burial rites of a person that dies of COVID-19
14. I don't know

15. Proper hand washing include the following? *(Do not read options, tick what is shown)*

1 Washing between the fingers
2 Washing the back of the hand
3 Washing the base of thumb
4 Washing the back of fingers
5 Washing the fingernails
6 Washing the palm

Sources and channels of information regarding COVID-19

16. How did you hear about COVID-19? *(Do not read options, tick what is mentioned. Multiple responses allowed)*

1. Radio
2. Television
3. Journal
4. Newspaper
5. Health Educator
6. Town announcer
7. Mosque
8. Church
9. Family member
10. Peers
11. Health facility
12. Flyer
13. Internet sites
14. Social media *(Facebook, Twitter, WhatsApp)*
15. GSM/SMS
16. Market
17. Neighbourhood
18. Others *(please state)*

Attitude and perception regarding COVID-19
**17. Do you think COVID-19 is a problem in Nigeria?**<br>1. Yes 2. No 3. I don't know<br>**17a. If no, specify** *(Do not read options, tick what is mentioned. Multiple responses allowed)*<br>**1.** It is just being exaggerated **2.** People just want to make money with COVID-19 drug<br>3. There are only few cases 4. I do not believe that there are cases of EVD<br>*5.* Other *(please state)*
**17b. If yes, specify** *(Do not read options, tick what is mentioned. Multiple responses allowed)*<br>**1.** It is a deadly disease **2.** It has no cure **3.** It is highly infectious<br>4. It is an attack by the Western world 5. It creates a lot of panic<br>6. Other *(please state)*
**18. Do you think you can contract COVID-19?**<br>1. Yes 2. No 3. I don't know<br>**18a. If no, why not?**
**19. Do you think government is doing enough to contain the COVID-19 outbreak?**<br>1. Yes 2. No 3 I don't know<br>**19a. If no, what other things do you think government should do to contain the COVID-19 outbreak?**
**Practices regarding COVID-19**
**20. What would you do if you develop signs of COVID-19?** *(Do not read options, tick what is mentioned. Multiple responses allowed)*<br>1. Go to the hospital 2. Call the COVID-19 help number 3. Go to a traditional healer<br>4. Stay at home 5. Treat myself *(specify type of treatment)*<br>6. Pray 7. Hide 8. Go to religious centre 9. Do nothing<br>*10.* Other *(please state)*
**21. If a family member, relative/neighbor develops signs of COVID-19, what advice will you give?** *(Do not read options, tick what is mentioned. Multiple responses allowed)*<br>1. Go to the hospital 2. Call the COVID-19 help number 3. Go to a traditional healer<br>4. Stay at home 5. Home treatment *(specify type of treatment)*<br>6. Pray 7. Hide 8. Go to religious centre 9. Do nothing<br>10. Other *(please state)*
**22. Is there anything you have stopped doing since the COVID-19 outbreak started?**<br>1. No 2. No *(if yes, please state)*
**23. Is there anything you have just started doing since the COVID-19 outbreak started?**<br>1. Yes 2. No *(if yes, please ask). Frequent hand washing 2. Social distancing 3. Use of face mask 4. cough etiquette 5. Others (specify)*
**24. Would you eat with a person with COVID-19 who has been treated and discharged?** 1 Yes 2 No
**25. Would you talk to a person with COVID-19 who has been treated and discharged?** 1 Yes 2 No
**26. Have you heard of the COVID-19 alert number?** 1 Yes 2 No
**26a.** ***(if yes)*** **What is it?**
**27. Have you heard of the COVID-19 alert website?** 1 Yes 2 No
**27a.** ***(if yes)*** **What is it?**
**28. Has COVID-19 affected you in any way? 1. Yes 2. No (If yes specify)**
**29. What do you think the government should do to control COVID-19?**

Thank you
